# Supplementary material for: Surface Microbiome Profiling of Dental Elevators Using Third‐Generation Sequencing: Implications for Infection Control in Dental Practice
Source: Microbiologyopen. 2025 Dec 8;14(6):e70178. doi: 10.1002/mbo3.70178 (PMC12685768; doi:10.1002/mbo3.70178)
Supplement: Supplementary file 1 — customer_backup. [file MBO3-14-e70178-s001.zip › customer_backup/customer_backup/function_analysis/BugBase/treat/otu_contributions/species/taxa_legend.pdf]

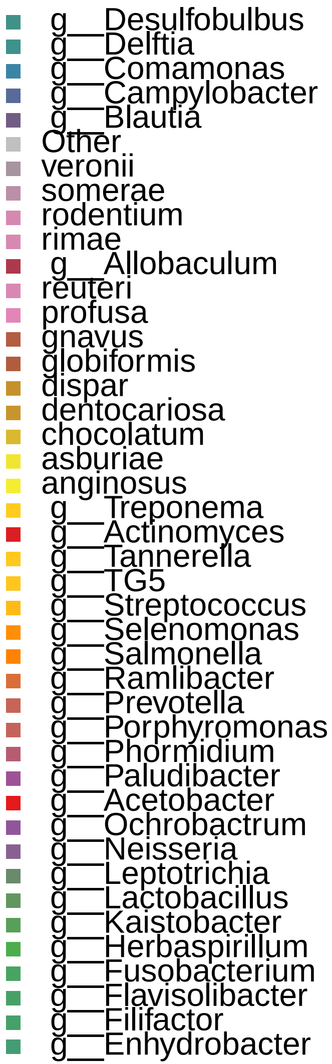
g__Desulfobulbus
g__Delftia
g__Comamonas
g__Campylobacter
g__Blautia
Other
veronii
somerae
rodentium
rimae
g__Allobaculum
reuteri
profusa
gnavus
globiformis
dispar
dentocariosa
chocolatum
asburiae
anginosus
g__Treponema
g__Actinomyces
g__Tannerella
g__TG5
g__Streptococcus
g__Selenomonas
g__Salmonella
g__Ramlibacter
g__Prevotella
g__Porphyromonas
g__Phormidium
g__Paludibacter
g__Acetobacter
g__Ochrobactrum
g__Neisseria
g__Leptotrichia
g__Lactobacillus
g__Kaistobacter
g__Herbaspirillum
g__Fusobacterium
g__Flavisolibacter
g__Filifactor
g__Enhydrobacter
